# The occurrence and co-occurrence of conflicts and negative acts and their associations with self-rated health, workability, and life-satisfaction: a cross-sectional study of Swedish school principals

**DOI:** 10.1186/s13104-025-07540-5

**Published:** 2025-10-20

**Authors:** Roger Persson, Ulf Leo, Carita Håkansson

**Affiliations:** 1 Department of Psychology, Lund University, Lund, Sweden; 2 Centre for Principal Development, Umeå university, Umeå, Sweden; 3 Division of Occupational and Environmental Medicine, Lund University, Lund, Sweden

**Keywords:** Bullying, Harassment, Sexual harassment, Threat, Violence

## Abstract

**Objective:**

As part of our research on Swedish school principals, we examined the occurrence and co-occurrence of conflicts and five negative acts (i.e. harassment, sexual harassment, threats of violence or physical harm, physical violence, bullying), their attributed sources, and their associations with self-rated health, workability, and general life satisfaction among 2670 principals.

**Results:**

During the past 12 months, approximately 75% of the school principals had experienced conflicts, 27.1% harassment, 22.4% threats of violence or physical harm, 7.2% physical violence, 5.9% bullying, and 2.7% sexual harassment. Some 18.9% reported neither involvement in conflicts nor being subjected to negative acts. Parents and teachers were common counterparts in conflicts and harassment. Parents and students made frequent threats of violence and physical harm. Mainly students carried out acts of violence. Logistic regression analyses showed that reports of being harassed, threatened, and bullied were consistently associated with lower self-rated health, workability, and general life satisfaction. School principals who had been subjected to sexual harassment reported lower workability in relation to psychological demands and lower general life satisfaction. School principals’ health, workability, and general life satisfaction may benefit from taking measures that ensure proper social interactions with both internal (e.g. superiors) and external stakeholders (e.g. parents).

**Supplementary Information:**

The online version contains supplementary material available at 10.1186/s13104-025-07540-5.

## Introduction

Due to the probability of negative consequences for individuals (e.g. poor health), organisations (e.g. reduced productivity, turnover), and society (e.g. sick leave, early retirement), the value of being attentive to emerging risks and broadly monitoring psychosocial hazards at work – such as violence, harassment, work-life imbalance, and work-related stress – has been acknowledged [1]. However, the working conditions of managers are often overlooked in research and in practice [2].

In Sweden, school principals are managers with an important societal mission that includes educating students and preparing them for life in society. They are also responsible for the internal organisation, pedagogical development, and the working environment of both teachers and students [3]. Since overburdened school principals may suffer reduced health and/or impaired function in their roles, we have previously examined the working conditions, mental health, and well-being of this occupational group [4–11]. Here, we extend this research by examining indicators of problematic social interactions and their associations with indicators of health, workability, and life satisfaction.

Cooperation is an important everyday task for school principals and a decisive organising principle in human societies [12, 13]. However, people often interact, intentionally or unintentionally, without considering the welfare of others. In work settings, problematic social interactions may among other things be manifested as conflicts, discrimination, harassment, bullying, ostracism, and violence. For example, a study based on the fourth European Working Conditions Survey (EWCS), which entailed >29.000 respondents from 31 countries, estimated that 7–8% of the workers were exposed to physical violence, 5–7% to bullying, 5–6% to discrimination, and 1–3% to sexual harassment [14]. Various forms of maltreatment (e.g. bullying, being yelled at, corporal punishment) involving numerous stakeholders (e.g. students, teachers, family) are phenomena also known to occur in schools across the world [15]. However, very few studies have examined and reported on school principals’ experiences of problematic social interactions or negative acts [16, 17]. Yet, one recent study, which reported pooled prevalence data (gathered annually between 2011 and 2019) showed that 36.2% of Australian principals and assistant principals had been subject to bullying and that 48.6% and 38.7% had been subjected to threats of violence or physical violence, respectively [16]. Also, a study from New Zeeland, which estimated the 12-month prevalence, showed that 22.9% of the participating school principals had been bullied and that 32.0% and 34.8% had been subject to threats or physical violence, respectively [17].

In Sweden, threats and violence within the school system has recently been reported to constitute a societal problem of current national interest [18, 19]. Since Swedish school principals have been expected to take on more responsibilities with even greater efficiency in recent decades [20], it is likely that the risk for experiencing misunderstandings, disputes, and social conflicts with both internal and external stakeholders has increased. For example, the Swedish Work Environment Authority report that threats and violence toward school employees has increased markedly [18] and the Swedish Schools Inspectorate have argued that an increasing influx of reports concerning mistreatment of students, teachers and/or principals motivate an increased budget [21].

To help authorities, employers, and other stakeholders to better understand Swedish school principals’ experiences of problematic social interactions and negative acts, assess associated risks, and take informed actions, we set out to investigate:

1. The occurrence of conflicts and negative acts in the form of harassment, sexual harassment, threats of violence or physical harm, physical violence, and bullying.
2. The co-occurrence of conflicts and the five negative acts.
3. How common it is for superiors, colleagues, teachers, other staff, students/children, caregivers, and other individuals to be counterparts in conflicts and/or negative acts involving school principals.
4. The strength of association between conflicts and negative acts and indicators for self-rated health, current workability, and general life satisfaction.

## Main text

### Methods

#### Participants and study design

The participants and the study design are identical to Persson et al. [11]. Details regarding the identification and selection of participants can be found in previous articles [7, 8, 21]. In brief, the first response from school principals who worked at least 50% of full-time in compulsory schools (46%), preschools (27%), upper secondary schools (15%), adult education (7%), or preschools and compulsory schools combined (5%), and who had completed an online survey issued in 2018 (*n* = 2219) and 2019 (*n* = 451) were included (*n* = 2670). The mean age was 49.4 years (SD = 7.3 years), 78% were women, and 77% were employed by a municipality. When compared with national data the study sample is reasonably representative as regards age and gender distribution [22, 23].

#### Measures

All data were self-reported. Background information used in the present analyses included *age*, *gender*, *school form* and *years of experience as a school leader*.

Six items, derived from a bi-annual Danish population study [24] and slightly modified to be contextually appropriate, were used to assessed conflicts and five negative acts (see Table S1). Responses were rated on a five-point scale: No, never; Yes, but not every month; Yes, monthly; Yes, every week; Yes, daily. In the questionnaire, all items were presented individually and the section header framed them all as concerning the workplace. They read:

*Have you*,* during the past 12 months…*,

1. *…had any* *conflicts* *with anybody at your workplace*?
2. *…been subjected to* *harassment* *at your workplace*?
3. …*been subjected to* *harassment of a sexual nature* *that has violated your dignity?*
4. *…been subjected to* *threats of violence or threats of physical harm* *at your workplace?*
5. …*been subjected* *to physical violence* *at your workplace?*
6. … *been* *bullied*? This item was preceded by a definition: “Bullying means a behaviour in which one or more people, over a longer period of time – regularly or several times – expose one or more other people to behaviour that they perceive as harmful or degrading”.

Each item was followed up by a question asking respondents to identify who they perceived as counterparts. Multiple answers were allowed, and the seven response categories were: *Superior*, *Manager colleague*, *Teacher*, *Other staff*, *Students/children*, *Parents/Caregivers*, *Other individuals*.

Self-rated health was assessed with the question, “*How do you assess your general state of health*” [25], and scored on a five-point scale: Poor; Fairly poor; Neither good nor poor; Fairly good; Very good. For purposes of analysis, this item was dichotomised into 0 = Fairly good to very good, and 1 = Poor to neither good nor poor.

Workability was assessed with items 1a, 2a, and 2b from the Work Ability Index (WAI) [26]. Considered a valid proxy in large-scale studies [27–29] item 1a read, “*If you compare your current workability with your lifetime best*,* what score would you give your current workability; we assume that your workability at its best is valued as 10 points*”, and was rated on an 11-step scale with verbal anchors at the endpoints (0 = Completely unable to work and 10 = Workability at its best). For purposes of analysis, drawing on other studies [6, 30], the score was also dichotomised into 0 = Excellent workability (9–10), and 1 = Poor to good workability (0–8).

Items 2a and 2b [26] assessed workability in relation to physical and psychological demands, respectively, and read “*How do you assess your current workability in relation to the* *physical/psychological* *demands of your job*?” Responses were rated on a five-point scale: Very poor; Rather poor; Moderate; Rather good; Very good. For purposes of analysis and in line with previous research [5], each item was dichotomised into 0 = Rather good and very good, and 1 = Moderate, rather poor, and very poor.

One item from the Life Satisfaction Questionnaire assessed life satisfaction: “*How satisfied are you with your life in general?*” Responses were rated on a six-point scale: Very dissatisfied; Dissatisfied; Pretty dissatisfied; Pretty satisfied; Satisfied; Very satisfied [31, 32]. For purposes of analysis, this item was dichotomised into 0 = Very satisfied to pretty satisfied, and 1 = Pretty dissatisfied to very dissatisfied.

#### Statistical analysis

IBM SPSS software (version 30) was used, and two-tailed p-values ≤ 0.05 were considered statistically significant. Spearman rank order correlations were used to estimate the strength of association between discrete, ordinal variables, and continuous variables. Chi-square tests of independence tested for differences across gender, school forms, and years of experience as a school principal. Logistic regression analyses were used to predict five binary outcomes for independent category variables that were derived from the six conflict/negative acts items. The five binary outcomes comprised self-rated health, workability (3 outcomes), and general life satisfaction. Results were expressed as prevalence odds ratios (OR) with accompanying 95% confidence intervals (CI).

### Results

Table 1 presents the prevalence proportions for conflicts, negative acts and their co-occurrence, and attributed sources. Prevalence proportions for subgroups defined by school form, gender, and length of work experience as a school principal are found in supplementary tables S2 and S3.

**Table 1:** Experienced conflicts and negative acts in the past 12 months and their attributed sources

	Conflicts		Harassment		Sexual-Harassment		Threats of violence and physical harm		Violence		Bullying	
Frequency of experience	*N*	%	*N*	%	*N*	%	*N*	%	*N*	%	*N*	%
Yes, daily	9	0.3	9	0.3	0	0.0	2	0.1	1	0.0	5	0.2
Yes, every week	132	4.9	34	1.3	2	0.1	9	0.3	5	0.2	18	0.7
Yes, every month	336	12.6	93	3.5	4	0.1	35	1.3	8	0.3	28	1.0
Yes, but not every month	1517	56.8	588	22.0	65	2.4	553	20.7	179	6.7	106	4.0
No, never	676	25.3	1946	72.9	2599	97.3	2071	77.6	2477	92.8	2513	94.1
Yes, total ^a^	1994	74.7	724	27.1	71	2.7	599	22.4	193	7.2	157	5.9
**Attributed counterparts** ^b^	**N** (1994)	**%**	**N** (724)	**%**	**N** (71)	**%**	**N** (599)	**%**	**N** (193)	**%**	**N** (157)	**%**
Superior	228	11.4	79	10.9	5	7.0	1	0.2	0	0.0	38	24.2
Manager colleague	252	12.6	67	9.3	12	16.9	2	0.3	0	0.0	45	28.7
Teacher	1022	51.3	139	19.2	12	16.9	15	2.5	1	0.5	53	33.8
Other staff than teacher	610	30.6	65	9.0	10	14.1	11	1.8	1	0.5	24	15.3
Students/children	330	16.5	105	14.5	11	15.5	291	48.6	185	95.9	8	5.1
Parents/caregivers	1040	52.2	434	59.9	16	22.5	318	53.1	10	5.2	16	10.2
Other persons	122	6.1	46	6.4	11	15.5	61	10.2	3	1.6	5	3.2
**Co-occurrence of conflicts and negative acts**												
**Conflicts and negative acts (6 items)**	**N**	**%**										
0	505	18.9	–	–	–	–	–	–	–	–	–	–
1	1180	44.2	–	–	–	–	–	–	–	–	–	–
2	546	20.4	–	–	–	–	–	–	–	–	–	–
3	316	11.8	–	–	–	–	–	–	–	–	–	–
4 or more	124	4.7	–	–	–	–	–	–	–	–	–	–
**Negative acts only (5 items)**	**N**	**%**										
0	1557	58.3	–	–	–	–	–	–	–	–	–	–
1	635	23.8	–	–	–	–	–	–	–	–	–	–
2	348	13.0	–	–	–	–	–	–	–	–	–	–
3	109	4.1	–	–	–	–	–	–	–	–	–	–
4 or more	21	0.8	–	–	–	–	–	–	–	–	–	–

Participants were allowed to provide multiple answers for attributed sources (*N* = 2670)

a = Prevalence proportion across all yes answers; b = N and % refer to the amount of yes answers that are observed within each of the six categories. Multiple answers were allowed, and the column percentages can therefore exceed 100%

In brief, over the past 12 months, 74.7% of the school principals had experienced conflicts, 27.1% harassment, 22.4% threats of violence or physical harm, 7.2% physical violence, 5.9% bullying, 2.7% sexual harassment, and 18.9% reported neither involvement in conflicts nor having been subjected to negative acts.

Parents/caregivers were the most common counterpart in conflicts, harassment, sexual harassment, and threats of violence and physical harm. Students were the most common counterpart in violence and a close second concerning threats of violence and physical harm. Teachers, followed by manager colleagues and superiors, were the most common counterparts concerning bullying (Table 1).

Table 2 presents descriptive mean scores and the correlations between scores for age, conflict, negative acts, self-rated health, workability, and general life satisfaction.

**Table 2:** Mean scores, standard deviations (SD), and spearman rank order correlations for age, self-rated health, indicators for workability, general life satisfaction and conflicts, and five negative acts (*N* = 2670)

	Mean	SD	1	2	3	4	5	6	7	8	9	10	11	12
1. Age (years)	49.4	7.3	1											
2. SRH 5	3.90	0.77	− 0.013	1										
3. WAI 1a	8.61	1.67	0.013	0.333^**^	1									
4. WAI 2a physical	4.43	0.69	− 0.058^**^	0.357^**^	0.378^**^	1								
5. WAI 2b psychological	3.83	0.87	0.052^**^	0.366^**^	0.602^**^	0.439^**^	1							
6. General Life satisfaction	4.64	1.05	0.043^*^	0.398^**^	0.375^**^	0.257^**^	0.406^**^	1						
7. Conflicts	1.98	0.78	0.016	*− 0.079* ^**^	*− 0.152* ^**^	*− 0.048* ^*^	*− 0.142* ^**^	*− 0.122* ^**^	1					
8. Harassment	1.34	0.64	0.040^*^	*− 0.080* ^**^	*− 0.112* ^**^	*− 0.078* ^**^	*− 0.154* ^**^	*− 0.133* ^**^	0.318^**^	1				
9. Sexual harassment	1.03	0.19	− 0.107^**^	*− 0.031*	*− 0.017*	*− 0.031*	*− 0.058* ^**^	*− 0.062* ^**^	0.063^**^	0.120^**^	1			
10. Threats	1.25	0.49	− 0.032	*− 0.084* ^**^	*− 0.067* ^**^	*− 0.072* ^**^	*− 0.090* ^**^	*− 0.079* ^**^	0.181^**^	0.313^**^	0.079^**^	1		
11. Violence	1.08	0.31	− 0.015	*− 0.047* ^*^	*− 0.022*	*− 0.006*	*− 0.052* ^**^	*− 0.033*	0.046^*^	0.097^**^	0.044^*^	0.297^**^	1	
12. Bullying	1.09	0.41	0.092^**^	*− 0.086* ^**^	*− 0.092* ^**^	*− 0.058* ^**^	*− 0.095* ^**^	*− 0.065* ^**^	0.161^**^	0.312^**^	0.078^**^	0.105^**^	0.024	1

*p < 0.05; **p < 0.001

Italics indicates the associations between outcome scores and conflicts and negative act scores

SRH 5 = Self-rated health 5 (1 = very poor; 5 = very good)

WAI 1a = Workability score, current workability compared with lifetime best (0 = completely unable to work; 10 = work ability at its best)

WAI 2a = Workability in relation to the physical demands of the job (1 = very poor; 5 = very good)

WAI 2b = Workability in relation to the physical demands of the job (1 = very poor; 5 = very good)

General Life Satisfaction (1 = very dissatisfied; 6 = very satisfied)

For conflicts and negative acts (1 = No, never; 2 = Yes, but more seldom than every month; 3 = Yes, monthly; 4 = Yes, every week; 5 = Yes, daily)

Table 3 presents unadjusted logistic regression analyses showing that experiences of conflicts in the past 12 months were associated with lower workability and general life satisfaction scores but not self-rated health scores. Experiences of being harassed, threatened, or bullied were consistently associated with reports of poorer health, and lower ratings of workability and general life satisfaction. Experiences of violence and sexual harassment over the past 12 months were associated with ratings of poor workability in relation to the psychological demands of the job, while sexual harassment was associated with lower general life satisfaction.

**Table 3:** Unadjusted bi-variate logistic regression analyses between conflicts, five negative acts, and three types of outcomes (*N* = 2670)

	Outcome 1: Self-rated health (1–5)			Outcome 2: Workability in relation to lifetime best (0–10)			Outcome 2a: Workability in relation to the physical demands (1–5)			Outcome 2b: Workability in relation to the psychological demands (1–5)			Outcome 3: General life satisfaction (1–6)		
	OR increase with 95% CI, Good, very good (= 0) Vs. Poor, neither good nor poor (= 1)			OR increase with 95% CI, Good, very good (= 0) Vs. Poor, neither good nor poor (= 1)			OR increase with 95% CI, Very good/fairly Good (= 0) Vs. Medium/fairly poor/very poor (= 1)			OR increase with 95% CI, Very good/fairly Good (= 0) Vs. Medium/fairly poor/very poor (= 1)			OR increase with 95% CI, Very satisfied to fairly satisfied (= 0) Vs. fairly unsatisfied to very unsatisfied (= 1)		
**Independent variables**	**OR**	**95% CI**	**P-value**	**OR**	**95% CI**	**P-value**	**OR**	**95% CI**	**P-value**	**OR**	**95% CI**	**P-value**	**OR**	**95% CI**	**P-value**
** Conflicts**															
Yes	1.17	0.94–1.45	0.166	1.66	1.38–1.99	< 0.001	1.40	1.00-1.96	0.049	1.70	1.38–2.09	< 0.001	1.47	1.09–1.98	0.011
No	1.0	–	–	1.0	–	–	1.0	–	–	1.0	–	–	1.0	–	–
** Harassment**															
Yes	1.43	1.17–1.75	< 0.001	1.42	1.17–1.72	< 0.001	1.67	1.26–2.21	< 0.001	1.83	1.53–2.20	< 0.001	1.80	1.40–2.31	< 0.001
No	1.0	–	–	1.0	–	–	1.0	–	–	1.0	–	–	1.0	–	–
** Sexual harassment**															
Yes	1.42	0.84–2.39	0.195	1.29	0.75–2.21	0.359	1.76	0.89–3.49	0.103	1.90	1.18–3.06	0.009	3.18	1.87–5.42	< 0.001
No	1.0	–	–	1.0	–	–	1.0	–	–	1.0	–	–	1.0	–	–
** Threats**															
Yes	1.40	1.13–1.73	0.002	1.37	1.11–1.68	0.003	1.40	1.03–1.89	0.030	1.45	1.19–1.76	< 0.001	1.67	1.29–2.17	< 0.001
No	1.0	–	–	1.0	–	–	1.0	–	–	1.0	–	–	1.0	–	–
** Violence**															
Yes	1.31	0.94–1.83	0.113	1.30	0.93–1.82	0.124	1.02	0.61–1.71	0.936	1.42	1.05–1.94	0.025	1.47	0.98–2.22	0.063
No	1.0	–	–	1.0	–	–	1.0	–	–	1.0	–	–	1.0	–	–
** Bullying**															
Yes	2.21	1.58–3.10	< 0.001	1.90	1.27–2.86	0.002	2.12	1.35–3.33	0.001	2.10	1.51–2.91	< 0.001	2.19	1.46–3.28	< 0.001
No	1.0	–	–	1.0	–	–	1.0	–	–	1.0	–	–	1.0	–	–
** Co-occurrence: all ** ** 6 items**															
3 or more	1.76	1.30–2.39	< 0.001	2.09	1.58–2.78	< 0.001	1.98	1.26–3.09	0.003	2.80	2.09–3.76	< 0.001	2.65	1.77–3.97	< 0.001
2	1.34	0.99–1.81	0.052	2.16	1.65–2.82	< 0.001	1.64	1.06–2.56	0.028	2.33	1.75–3.10	< 0.001	1.71	1.13–2.57	0.010
1	1.02	0.78–1.33	0.896	1.36	1.09–1.69	0.006	1.06	0.70–1.60	0.784	1.47	1.13–1.90	0.004	1.23	0.84–1.79	0.284
0	1.0	–	–	1.0	–	–	1.0	–	–	1.0	–	–	1.0	–	–
** Co-occurrence: 5 ** ** negative acts items**															
3 or more	1.96	1.33–2.90	< 0.001	1.59	1.05–2.40	0.028	2.53	1.53–4.20	< 0.001	2.51	1.74–3.62	< 0.001	3.59	2.34–5.50	< 0.001
2	1.67	1.28–2.18	< 0.001	1.63	1.25–2.13	< 0.001	1.52	1.02–2.26	0.039	1.95	1.52–2.50	< 0.001	1.78	1.26–2.50	< 0.001
1	1.31	1.05–1.63	0.019	1.53	1.24–1.88	< 0.001	1.47	1.07–2.04	0.018	1.60	1.31–1.96	< 0.001	1.44	1.07–1.93	0.015
0	1.0	–	–	1.0	–	–	1.0	–	–	1.0	–	–	1.0	–	–

Having experienced at least one or more of the five negative acts over the past 12 months were consistently, and often incrementally, associated with reports of poor self-rated health, lower workability and lower general life satisfaction. When the conflict item was included in the calculations, the results changed slightly, although 26 of the 30 estimations retained a p-value < 0.05 (Table 3).

Adjusting for age, school form and gender did not change the results from the logistic regression analysis (Table S4).

### Discussion

The finding that 75% of the school principals experienced a conflict at least once in the past 12 months is considerably higher than the 18% to 22% reported by employees and managers in the recurring Danish population study from which these questions were derived [24]. Notably, most conflicts (56%) were reported to occur more seldom than every month. It is conceivable that this reflects an inherent feature in school leadership that not necessarily is negative. Indeed, it may be argued that part of the job description of being a school principal, or manager, entails dealing with conflicts and they may as such be experienced as something normal and stimulating. However, it cannot be determined whether the large difference in prevalence proportions between the Danish and Swedish study samples is due to the school principals’ unique working conditions or cultural and/or linguistic differences.

Comparisons across studies and countries are difficult to make due to variations in definitions, measurements, and cultural perceptions. However, the prevalence proportions for the five negative acts seem roughly comparable with international estimates. For example, in a sample representative of employed and self-employed people in 31 European countries, 7–8% reported exposure to physical violence, 5–7% to bullying and 1–3% sexual harassment [14]. In the bi-annual Danish studies [24, 33], approximately 11% reported being bullied, approximately 8% reported being exposed to threats, approximately 6% reported exposure to violence, and 2 to 4% sexual harassment. When compared with a mixed group of Danish managers [24, 33], the school principals reported noticeably more threats of violence and physical harm (22% vs. 3.7 to 6.0%, respectively), actual violence (7% vs. 1.2 to 2.3%, respectively), slightly higher levels of sexual harassment (2.7% vs. 0.9 to 1.5%, respectively) but similar levels of bullying (5.9% vs. 5.5 to 7.2%, respectively). Noticeably, both Danish managers and Swedish school principals report a lower occurrence of bullying, threats, and actual violence than school principals in New Zeeland who reported a 12-month prevalence of 22.9%, 32.0% and 34.8%, respectively [17].

The school principals primarily reported conflicts with parents, teachers, and other staff members, which suggests that school principals are engaged in both external and internal conflicts with ostensibly different origins. Parents/caregivers were the most reported counterpart in harassment, sexual harassment, and threats of violence and physical harm. The fact that primarily parents/caregivers and students threatened school principals, and mainly students resorted to violence, corresponds with a recent analysis by the Swedish Work Environment Authority examining formal reports of threats and violence targeting teachers and other staff [18, 19]. That students are reported to be the primary source of violent acts, and that both students and parents are reported to be common sources of threats of violence or physical harm, is also in agreement with previous observations from New Zeeland [17] and Australia [16]. Anyhow, the more dispersed reporting of counterparts among principals who experienced sexual harassment and bullying suggests that efforts to prevent these events may be more resource-intensive and difficult to implement.

The fact that conflict and negative act scores, both when assessed individually or in concert, often were associated with self-rated health, current workability, and general life satisfaction scores appears to confirm their relevance as potential psychosocial risk factors affecting individual, organisational, and societal outcomes.

### Conclusions

Swedish school principals are not immune to conflicts or negative acts. Conflicts were common on a yearly basis, and approximately 40% of the school principals had experienced at least one of the five negative acts in the past 12 months. Reports of being harassed, threatened, and bullied were consistently associated with reports of reduced self-rated health, lower workability, and general life dissatisfaction. The pattern of results suggests that school principals’ health, work performance, and general life satisfaction may benefit from taking measures that ensure that the social interactions with both internal (e.g. students and teachers) and external stakeholders (e.g. parents) are timely, respectful, clear, and constructive so to avoid taking the form of unwanted conflicts and negative acts.

### Limitations

While the survey data provides insight into the school principals’ perceptions of being involved in conflicts and/or subjected to negative acts, it does not provide insight into the complexities of the underlying social dynamics. Furthermore, recent developments in Sweden concerning the increase in threats and violence in the school system suggest that prevalence proportions based on data from 2018 to 2019 should be interpreted with caution and that authorities, employers, and other stakeholders need consider the time lag when taking informed actions. The cross-sectional study design implies the possibility of reverse causality.

## Supplementary Information

Below is the link to the electronic supplementary material.

Supplementary Material 1.

Supplementary Material 2.

## Data Availability

Consistent with the approved ethical protocols, anonymised data are stored locally at Lund University, Lund, Sweden. Because the participants (in accordance with the approved study protocol) were guaranteed that the raw data would not be published on the Internet, access to data will only be granted to eligible researchers wanting to audit our research. Requests should be directed to the corresponding author.

## Abbreviation

CI: Confidence interval
