# Supplementary material for: The occurrence and co-occurrence of conflicts and negative acts and their associations with self-rated health, workability, and life-satisfaction: a cross-sectional study of Swedish school principals
Source: BMC Res Notes. 2025 Oct 20;18:436. doi: 10.1186/s13104-025-07540-5 (PMC12538727; doi:10.1186/s13104-025-07540-5)
Supplement: Supplementary file 1 — Supplementary Material 1. [file 13104_2025_7540_MOESM1_ESM.docx]

**Additional file 1: Supplementary Table S1**

**Table S1.** Overview of the conflict item and the five negative acts items used in the electronic questionnaire. The items were originally derived from a bi-annual Danish population study [24] and slightly modified to be contextually appropriate. Translated from Danish to Swedish to English by the researchers.

| **English** | **Swedish** | **Danish (original survey items)** |
| --- | --- | --- |
| Have you, during the past 12 months, had any conflicts with anybody at your workplace? | Har du, under de senaste 12 månaderna, haft några konflikter med någon på din arbetsplats? | Har du inden for de sidste 12 måneder haft skænderier eller konflikter med nogen på din arbejdsplads? |
| - Yes, daily. - Yes, every week - Yes, monthly - Yes, but not every month - No, never | - Ja, dagligen - Ja, varje vecka - Ja, varje månad - Ja, men mer sällan än varje månad - Nej, aldrig | - Ja, dagligt - Ja, ugenligt - Ja, månedligt - Ja, sjældnere - Nej, aldrig |
| If yes, with whom did you have conflicts?  You can select multiple options.   - Superior - Manager colleague - Teacher - Other staff - Students/children - Parents/Caregivers - Other individuals | Om ja, med vem hade du konflikter med?  Du kan välja flera alternativ   - Överordnad person - Chefskollega - Lärare - Annan personal än lärare - Barn/Elev (-er) - Förälder (vårdnadshavare) - Annan/andra personer | Hvis ja, hvem havde du skænderier eller konflikter med?  Sæt gerne flere krydser:   - Kolleger - Ledere - Underordnede - Kunder, klienter, patienter, elever m.fl. |
|  |  |  |
| Have you, during the past 12 months, been subjected to harassment at your workplace?  *Harassment is behavior that violates someone's dignity.* | Har du, under de senaste 12 månaderna, blivit utsatt för någon form av trakasserier på din arbetsplats?  *Trakasserier är uppträdande som kränker någons värdighet.* | *Not in the original survey.* |
| - Yes, daily. - Yes, every week - Yes, monthly - Yes, but not every month - No, never | - Ja, dagligen - Ja, varje vecka - Ja, varje månad - Ja, men mer sällan än varje månad - Nej, aldrig | *This item was derived from the sexual harassment item to allow a separation between harassment and sexual harassment. See sexual harassment item on page 2.* |
| If yes, who harassed you?  You can select multiple options.   - Superior - Manager colleague - Teacher - Other staff - Students/children - Parents/Caregivers - Other individuals | Om ja, med vem hade du konflikter med?  Du kan välja flera alternativ   - Överordnad person - Chefskollega - Lärare - Annan personal än lärare - Barn/Elev (-er) - Förälder (vårdnadshavare) - Annan/andra personer |  |

*Table S1 continues*

***Table S1 continued***

| **English** | **Swedish** | **Danish (original)** |
| --- | --- | --- |
| Have you, during the past 12 months, been subjected to harassment of a sexual nature that has violated your dignity? | Har du, under de senaste 12 månaderna, blivit utsatt för uppträdande av sexuell natur som kränkt din värdighet? | Har du inden for de sidste 12 måneder været udsat for seksuel chikane på din arbejdsplads? |
| - Yes, daily. - Yes, every week - Yes, monthly - Yes, but not every month - No, never | - Ja, dagligen - Ja, varje vecka - Ja, varje månad - Ja, men mer sällan än varje månad - Nej, aldrig | - Ja, dagligt - Ja, ugenligt - Ja, månedligt - Ja, sjældnere - Nej, aldrig |
| If yes, who did it to you?  You can select multiple options.   - Superior - Manager colleague - Teacher - Other staff - Students/children - Parents/Caregivers - Other individuals | Om ja, vem utsatte dig?  Du kan välja flera alternativ   - Överordnad person - Chefskollega - Lärare - Annan personal än lärare - Barn/Elev (-er) - Förälder (vårdnadshavare) - Annan/andra personer | Hvis ja, hvem udsatte dig for sexuel chikane?  Sæt gerne flere krydser:   - Kolleger - Ledere - Underordnede - Kunder, klienter, patienter, elever m.fl. |
|  |  |  |
| Have you, during the past 12 months, been subjected to THREATS of violence or TREATHS of physical harm at your workplace? | Har du, under de senaste 12 månaderna, blivit utsatt för HOT om våld eller HOT om fysisk skada på din arbetsplats? | Har du inden for de sidste 12 måneder været udsat for trusler om vold på din arbejdsplads? |
| - Yes, daily. - Yes, every week - Yes, monthly - Yes, but not every month - No, never | - Ja, dagligen - Ja, varje vecka - Ja, varje månad - Ja, men mer sällan än varje månad - Nej, aldrig | - Ja, dagligt - Ja, ugenligt - Ja, månedligt - Ja, sjældnere - Nej, aldrig |
| If yes, who threatened you?  You can select multiple options.   - Superior - Manager colleague - Teacher - Other staff - Students/children - Parents/Caregivers - Other individuals | Om ja, vem hotade dig?  Du kan välja flera alternativ   - Överordnad person - Chefskollega - Lärare - Annan personal än lärare - Barn/Elev (-er) - Förälder (vårdnadshavare) - Annan/andra personer | Hvis ja, hvem udsatte dig for trusler om vold?  Sæt gerne flere krydser:   - Kolleger - Ledere - Underordnede - Kunder, klienter, patienter, elever m.fl. |

*Table S1 continues*

***Table S1 continued***

| **English** | **Swedish** | **Danish (original)** |
| --- | --- | --- |
| Have you, during the past 12 months, been subjected to physical violence at your workplace? | Har du, under de senaste 12 månaderna, blivit utsatt för fysiskt våld på din arbetsplats? | Har du inden for de sidste 12 måneder været udsat for fysisk vold på din arbejdsplads? |
| - Yes, daily. - Yes, every week - Yes, monthly - Yes, but not every month - No, never | - Ja, dagligen - Ja, varje vecka - Ja, varje månad - Ja, men mer sällan än varje månad - Nej, aldrig | - Ja, dagligt - Ja, ugenligt - Ja, månedligt - Ja, sjældnere - Nej, aldrig |
| If yes, who subjected you to violence?  You can select multiple options.   - Superior - Manager colleague - Teacher - Other staff - Students/children - Parents/Caregivers - Other individuals | Om ja, vem utsatte dig för våldet?  Du kan välja flera alternativ   - Överordnad person - Chefskollega - Lärare - Annan personal än lärare - Barn/Elev (-er) - Förälder (vårdnadshavare) - Annan/andra personer | Hvis ja, hvem udsatte dig for fysisk vold?  Sæt gerne flere krydser:   - Kolleger - Ledere - Underordnede - Kunder, klienter, patienter, elever m.fl. |
|  |  |  |
| Have you, during the past 12 months been bullied?  *Bullying means a behaviour in which one or more people, over a longer period of time – regularly or several times – expose one or more other people to behaviour that they perceive as harmful or degrading.* | Har du, under de senaste 12 månaderna, blivit mobbad?  *Med mobbning menas ett uppträdande där en eller flera personer under en längre tidsperiod – regelbundet eller flera gånger – utsätter en eller flera andra personer för beteenden som de uppfattar som skadliga eller förnedrande.* | Har du inden for de sidste 12 måneder været udsat for mobning på din arbejdsplads?  *(Dvs., når én eller flere personer regelmæssigt og over længere tid - eller gentagne gange på grov vis - udsætter én eller flere andre personer for krænkende handlinger, som vedkommende opfatter som sårende eller nedværdigende).* |
| - Yes, daily. - Yes, every week - Yes, monthly - Yes, but not every month - No, never | - Ja, dagligen - Ja, varje vecka - Ja, varje månad - Ja, men mer sällan än varje månad - Nej, aldrig | - Ja, dagligt - Ja, ugenligt - Ja, månedligt - Ja, sjældnere - Nej, aldrig |
| If yes, who subjected you to bullying?  You can select multiple options.   - Superior - Manager colleague - Teacher - Other staff - Students/children - Parents/Caregivers - Other individuals | Om ja, vem mobbade dig?  Du kan välja flera alternativ   - Överordnad person - Chefskollega - Lärare - Annan personal än lärare - Barn/Elev (-er) - Förälder (vårdnadshavare) - Annan/andra personer | Hvis ja, hvem udsatte dig for mobning?  Sæt gerne flere krydser:   - Kolleger - Ledere - Underordnede - Kunder, klienter, patienter, elever m.fl. |
