# Supplementary material for: The occurrence and co-occurrence of conflicts and negative acts and their associations with self-rated health, workability, and life-satisfaction: a cross-sectional study of Swedish school principals
Source: BMC Res Notes. 2025 Oct 20;18:436. doi: 10.1186/s13104-025-07540-5 (PMC12538727; doi:10.1186/s13104-025-07540-5)
Supplement: Supplementary file 2 — Supplementary Material 2. [file 13104_2025_7540_MOESM2_ESM.docx]

**Additional file 2: Supplementary Tables S2, S3 and S4**

**Table S2.** Distribution of reports of experiences of conflicts and negative acts at least once in the past 12 months across school forms and gender (N=2670).

|  | **School form** | | | | | | | | | |  |  | **Gender*** | | | |  |
| --- | --- | --- | --- | --- | --- | --- | --- | --- | --- | --- | --- | --- | --- | --- | --- | --- | --- |
|  | **Preschool** | | **Preschool and compulsory school** | | **Compulsory school** | | **Upper secondary education** | | **Adult**  **Education** | | **Chi-2 test** |  | **Women** | | **Men** | | **Chi-2 test** |
|  | (N=727) | | (N=130) | | (N=1217) | | (N=407) | | (N=181) | |  |  | **(N=2082)** | | **(N=582)** | |  |
|  | **N** | **%** | **N** | **%** | **N** | **%** | **N** | **%** | **N** | **%** | ***P*** |  | ***N*** | ***%*** | ***N*** | ***%*** | ***P*** |
| **Conflicts** |  |  |  |  |  |  |  |  |  |  |  |  |  |  |  |  |  |
| Yes | 506 | **69.6_a_** | 107 | 77.5_a.b_ | 934 | 76.7_b_ | 315 | 77.4_b.c_ | 132 | 72.9_a.b_ | 0.004 |  | 1534 | 73.7_a_ | 454 | **78.0_b_** | .034 |
| No | 221 | 30.4_a_ | 31 | 22.5_a.b_ | 283 | 23.3_b_ | 92 | 22.6_b.c_ | 49 | 27.1_a.b_ |  |  | 548 | 26.3_a_ | 128 | 22.0_b_ |  |
| **Harassment** |  |  |  |  |  |  |  |  |  |  |  |  |  |  |  |  |  |
| Yes | 136 | 18.7_a_ | 37 | 26.8_a.b_ | 415 | **34.1_b_** | 97 | 23.8_a_ | 39 | 21.5_a_ | <.001 |  | 565 | **27.1_a_** | 155 | 26.6_a_ | .808 |
| No | 591 | 81.3_a_ | 101 | 73.2_a.b_ | 802 | 65.9_b_ | 310 | 76.2_a_ | 142 | 78.5_a_ |  |  | 1517 | 72.9_a_ | 427 | 73.4_a_ |  |
| **Sexual harassment** |  |  |  |  |  |  |  |  |  |  |  |  |  |  |  |  |  |
| Yes | 13 | 1.8 | 7 | **5.1** | 25 | 2.1 | 18 | 4.4 | 8 | 4.4 | NC |  | 57 | **2.7_a_** | 14 | 2.4_a_ | .660 |
| No | 714 | 98.2 | 131 | 94.9 | 1192 | 97.9 | 389 | 95.6 | 173 | 95.6 |  |  | 2025 | 97.3_a_ | 568 | 97.6_a_ |  |
| **Threat** |  |  |  |  |  |  |  |  |  |  |  |  |  |  |  |  |  |
| Yes | 89 | 12.2_a_ | 28 | 20.3_a.b.c_ | 367 | **30.2_b_** | 82 | 20.1_c_ | 33 | 18.2_a.c_ |  |  | 463 | 22.2_a_ | 134 | **23.0_a_** | .688 |
| No | 638 | 87.8_a_ | 110 | 79.7_a.b.c_ | 850 | 69.8_b_ | 325 | 79.9_c_ | 148 | 81.8_a.c_ | <.001 |  | 1619 | 77.8_a_ | 448 | 77.0_a_ |  |
| **Violence** |  |  |  |  |  |  |  |  |  |  |  |  |  |  |  |  |  |
| Yes | 14 | 1.9_a_ | 8 | 5.8_a.b_ | 156 | **12.8_b_** | 11 | 2.7_a_ | 4 | 2.2_a_ |  |  | 151 | **7.3_a_** | 41 | 7.0_a_ | .864 |
| No | 713 | 98.1_a_ | 130 | 94.2_a.b_ | 1061 | 87.2_b_ | 396 | 97.3_a_ | 177 | 97.8_a_ | <.001 |  | 1931 | 92.7_a_ | 541 | 93.0_a_ |  |
| **Bullying** |  |  |  |  |  |  |  |  |  |  |  |  |  |  |  |  |  |
| Yes | 33 | 4.5 | 10 | 7.2 | 72 | 5.9 | 27 | 6.6 | 15 | **8.3** |  |  | 137 | **6.6_a_** | 20 | 3.4 _b_ | .004 |
| No | 694 | 95.5 | 128 | 92.8 | 1145 | 94.1 | 380 | 93.4 | 166 | 91.7 | 0.273 |  | 1945 | 93.4_a_ | 562 | 96.6 _b_ |  |

Note: Values in the same row and sub-table not sharing the same subscript are significantly different at p < 0.05 in the two-sided test of equality for column proportions. Tests are adjusted for all pairwise comparisons within a row of each innermost sub-table using the Bonferroni correction. For navigational purposes, **bold** figures indicate, irrespective of statistical significance, the lowest OR highest observed value across the individual school forms for conflict and the negative act categories. NC = Not calculated due to too few observations across the individual cells. *Six individuals did not disclose gender.

**Table S3.** Distribution of reports of experiences of conflicts and negative acts at least once in the past 12 months across years of experience as a school principal (N=2670).

|  | **Up to 3 years** | | **> 3 to 5 years** | | **> 5 to 10 years** | | **>10 years or more** | |  |  |
| --- | --- | --- | --- | --- | --- | --- | --- | --- | --- | --- |
|  | (N=472) | | (N=601) | | (N=944) | | (N=653) | | **Chi-2 test** |  |
|  |  | |  | |  | |  | |  |  |
|  | **N** | **%** | **N** | **%** | **N** | **%** | **N** | **%** | ***P*** |  |
| **Conflicts** |  |  |  |  |  |  |  |  |  |  |
| Yes | 353 | 74.8 | 444 | 73.9 | 706 | 74.8 | 491 | **75.2** | .959 |  |
| No | 119 | 25.2 | 157 | 26.1 | 238 | 25.2 | 162 | 24.8 |  |  |
| **Harassment** |  |  |  |  |  |  |  |  |  |  |
| Yes | 106 | 22.5 | 166 | 27.6 | 257 | 27.2 | 195 | **29.9** | .051 |  |
| No | 366 | 77.5 | 435 | 72.4 | 687 | 72.8 | 458 | 70.1 |  |  |
| **Sexual harassment** |  |  |  |  |  |  |  |  |  |  |
| Yes | 15 | 3.2 | 22 | **3.7** | 25 | 2.6 | 9 | 1.4 | .073 |  |
| No | 457 | 96.8 | 579 | 96.3 | 919 | 97.4 | 644 | 98.6 |  |  |
| **Threat** |  |  |  |  |  |  |  |  |  |  |
| Yes | 96 | 20.3 | 134 | 22.3 | 217 | 23.0 | 152 | **23.3** | .653 |  |
| No | 376 | 79.7 | 467 | 77.7 | 727 | 77.0 | 501 | 76.7 |  |  |
| **Violence** |  |  |  |  |  |  |  |  |  |  |
| Yes | 44 | **9.3** | 42 | 7.0 | 61 | 6.5 | 46 | 7.0 | .262 |  |
| No | 428 | 90.7 | 559 | 93.0 | 883 | 93.5 | 607 | 93.0 |  |  |
| **Bullying** |  |  |  |  |  |  |  |  |  |  |
| Yes | 29 | 6.1 | 34 | 5.7 | 53 | 5.6 | 41 | **6.3** | .421 |  |
| No | 443 | 93.9 | 567 | 94.3 | 891 | 94.4 | 612 | 93.7 |  |  |

Note: For navigational purposes, **bold** figures indicate, irrespective of statistical significance, the lowest OR highest observed value across the length of experience categories.

**Table S4.** Estimates from age and school form adjusted* logistic regression analyses between conflicts and negative acts and three types of outcomes (N=2670)

|  | **Outcome 1:**  **Self-rated health (1-5)** | | | **Outcome 2:**  **Workability in relation to lifetime best (0-10)** | | | **Outcome 2a:**  **Workability in relation to the physical demands (1-5)** | | | **Outcome 2b:**  **Workability in relation to the psychological demands (1-5)** | | | **Outcome 3:**  **General life satisfaction (1-6)** | | |
| --- | --- | --- | --- | --- | --- | --- | --- | --- | --- | --- | --- | --- | --- | --- | --- |
|  | OR increase with 95% CI,  Good, very good (=0)  Vs. Poor, neither good nor poor (=1) | | | OR increase with 95% CI,  Good, very good (=0)  Vs. Poor, neither good nor poor (=1) | | | OR increase with 95% CI,  Very good/fairly Good (=0)  Vs. Medium/fairly poor/very poor (=1) | | | OR increase with 95% CI,  Very good/fairly Good (=0)  Vs. Medium/fairly poor/very poor (=1) | | | OR increase with 95% CI, Very satisfied to fairly satisfied (=0) Vs. fairly unsatisfied to very unsatisfied (=1) | | |
| **Independent variables** |  |  |  |  |  |  |  |  |  |  |  |  |  |  |  |
|  | **OR** | **95% CI** | P-value | **OR** | **95% CI** | P-value | **OR** | **95% CI** | P-value | **OR** | **95% CI** | P-value | **OR** | **95% CI** | P-value |
| **Conflicts** |  |  |  |  |  |  |  |  |  |  |  |  |  |  |  |
| Yes | 1.16 | 0.94-1.44 | 0.173 | 1.68 | 1.40-2.02 | <0.001 | 1.45 | 1.03-2.03 | 0.031 | 1.73 | 1.40-2.13 | <0.001 | 1.45 | 1.07-1.96 | 0.015 |
| No | 1.0 | -- | -- | 1.0 | -- | -- | 1.0 | -- | -- | 1.0 | -- | -- | 1.0 | -- | -- |
| **Harassment** |  |  |  |  |  |  |  |  |  |  |  |  |  |  |  |
| Yes | 1.43 | 1.17-1.75 | <0.001 | 1.45 | 1.19-1.76 | <0.001 | 1.81 | 1.36-2.41 | <0.001 | 1.88 | 1.56-2.26 | <0.001 | 1.78 | 1.38-2.29 | <0.001 |
| No | 1.0 | -- | -- | 1.0 | -- | -- | 1.0 | -- | -- | 1.0 | -- | -- | 1.0 | -- | -- |
| **Sexual Harassment** |  |  |  |  |  |  |  |  |  |  |  |  |  |  |  |
| Yes | 1.45 | 0.85-2.47 | 0.171 | 1.29 | 0.75-2.23 | 0.358 | 1.78 | 0.89-3.56 | 0.105 | 1.84 | 1.14-2.99 | 0.013 | 3.16 | 1.84-5.43 | <0.001 |
| No | 1.0 | -- | -- | 1.0 | -- | -- | 1.0 | -- | -- | 1.0 | -- | -- | 1.0 | -- | -- |
| **Threats** |  |  |  |  |  |  |  |  |  |  |  |  |  |  |  |
| Yes | 1.41 | 1.13-1.74 | 0.002 | 1.40 | 1.13-1.72 | 0.002 | 1.54 | 1.13-2.10 | 0.006 | 1.46 | 1.20-1.77 | <0.001 | 1.63 | 1.25-2.13 | <0.001 |
| No | 1.0 | -- | -- | 1.0 | -- | -- | 1.0 | -- | -- | 1.0 | -- | -- | 1.0 | -- | -- |
| **Violence** |  |  |  |  |  |  |  |  |  |  |  |  |  |  |  |
| Yes | 1.29 | 0.92-1.82 | 0.143 | 1.34 | 0.95-1.88 | 0.094 | 1.16 | 0.68-1.97 | 0.591 | 1.43 | 1.04-1.96 | 0.026 | 1.42 | 0.93-2.15 | 0.105 |
| No | 1.0 | -- | -- | 1.0 | -- | -- | 1.0 | -- | -- | 1.0 | -- | -- | 1.0 | -- | -- |
| **Bullying** |  |  |  |  |  |  |  |  |  |  |  |  |  |  |  |
| Yes | 2.22 | 1.58-3.12 | <0.001 | 1.94 | 1.29-2.91 | 0.002 | 2.17 | 1.37-3.43 | <0.001 | 2.22 | 1.60-3.09 | <0.001 | 2.16 | 1.44-3.25 | <0.001 |
| No | 1.0 | -- | -- | 1.0 | -- | -- | 1.0 | -- | -- | 1.0 | -- | -- | 1.0 | -- | -- |
|  |  |  |  |  |  |  |  |  |  |  |  |  |  |  |  |

Note: *Adjusting for gender, age, and school-form generated highly similar estimates compared with the unadjusted models as well as the here presented models (i.e., adjusted for age and school form).
